# Integrated analysis of microRNA-target interactions with clinical outcomes for cancers

**DOI:** 10.1186/1755-8794-7-S1-S10

**Published:** 2014-05-08

**Authors:** Je-Gun Joung, Dokyoon Kim, Su Yeon Lee, Hwa Jung Kang, Ju Han Kim

**Affiliations:** 1 Seoul National University Biomedical Informatics (SNUBI), Div. of Biomedical Informatics, Seoul National University College of Medicine, Seoul 110-799, Korea; 2 Systems Biomedical Informatics National Core Research Center, Seoul National University College of Medicine, Seoul 110-799, Korea; 3 Institute of Endemic Diseases, Seoul National University College of Medicine, 103 Daehakro, Jongno-gu, Seoul 110-799, Korea; 4 Center for Systems Genomics, Pennsylvania State University, University Park, Pennsylvania, USA; 5 Translational Bioinformatics Lab., Samsung Genome Institute, Samsung Medical Center, Seoul Korea

**Keywords:** Survival Analysis, miRNA-mRNA interaction, miRNA Expression, Gene Expression Profile, Cancer Genes

## Abstract

**Background:**

Clinical statement alone is not enough to predict the progression of disease. Instead, the gene expression profiles have been widely used to forecast clinical outcomes. Many genes related to survival have been identified, and recently miRNA expression signatures predicting patient survival have been also investigated for several cancers. However, miRNAs and their target genes associated with clinical outcomes have remained largely unexplored.

**Methods:**

Here, we demonstrate a survival analysis based on the regulatory relationships of miRNAs and their target genes. The patient survivals for the two major cancers, ovarian cancer and glioblastoma multiforme (GBM), are investigated through the integrated analysis of miRNA-mRNA interaction pairs.

**Results:**

We found that there is a larger survival difference between two patient groups with an inversely correlated expression profile of miRNA and mRNA. It supports the idea that signatures of miRNAs and their targets related to cancer progression can be detected via this approach.

**Conclusions:**

This integrated analysis can help to discover coordinated expression signatures of miRNAs and their target mRNAs that can be employed for therapeutics in human cancers.

## Background

As patterns of gene expression correlate with disease phenotype and patient outcome, mRNA expression profiling has been used to classify disease risks as well as prediction of outcome[1-4]. In addition, survival analysis with gene expression profiles is beneficial for identifying new prognostic targets of diverse diseases.

Many miRNAs have been also found to be correlated with clinical outcome in specific cancer types [5-10]. miRNAs are a class of small and endogenous RNA molecules that regulate their target mRNAs through translational repression or mRNA degradation [11]. In tumors, many miRNAs can be aberrantly expressed, leading to potentially abnormal regulation of their target mRNAs. Although over 1,000 human miRNAs may be encoded in human genome, the potential therapeutic markers provided by miRNAs for a diverse spectrum of diseases are still unexplored.

Recently, several investigations have put emphasis on the integrated analysis of miRNAs and mRNAs in clinical outcomes [12-17]. In general, there are different approaches for the joint analysis of miRNA and mRNA data. For example, several miRNAs associated with survival rate can be extracted by survival analysis and then their relationship of inverse correlation can be identified based on analyzing miRNA and mRNA expression profiles. Most approaches do not test the clinical outcome by considering miRNA expression and mRNAs expression simultaneously. At minimum, this analysis requires the size of the cohort to be large enough for statistically significant measurement outcomes and the paired samples with both mRNA and miRNA expressions should be given. The Cancer Genome Atlas (TCGA) [18] provides different types of genomic datasets and we systematically integrated multi-omics data for cancer clinical outcome prediction [19].

Here, we present the survival analysis considering the regulatory relationships of miRNAs and their target genes. We tested clinical outcomes with the patient survival information, miRNA expression profiles and gene expression profiles for ovarian cancer and glioblastoma multiforme (GBM) through the integrated analysis of miRNA-mRNA interaction pairs. We found the miRNA-mRNA pairs with an inversely correlated expression profile that have significant survival differences between two patient groups. The results presented here suggest that this analysis can help to discover expression signatures of miRNAs and their target mRNAs that can be employed for therapeutics in human cancers.

## Methods

### miRNA target dataset

We obtained the largest collection of human miRNA-mRNA target relationships from the TarBase 6.0 [20]. All targets in this collection were manually curated and experimentally validated. We extracted a total of 12,879 interactions consisting of 229 miRNAs and 6,699 target genes.

### Gene and miRNA expression profile

The miRNA and gene expression data sets in ovarian cancer and GBM are acquired from the Cancer Genome Atlas (TCGA) [18]. Ovarian cancer and GBM data sets consist of mRNA expression profiles and miRNA expression profiles for 496 and 425 patients, respectively. We transformed the expression values into a Z-score for each miRNA or each gene. If the Z-score of miRNA (or mRNA) is greater (less) than zero, its expression level is defined to be high (low). We excluded miRNA-mRNA pairs from the expression datasets that were not listed in the TarBase in order to avoid unnecessary calculations. Expression matrices for ovarian cancer composed of 137 miRNAs × 496 patients and 5,707 target genes × 496 patients. Among all possible 776,016 miRNAs and mRNA pairs, the number of validated interactions presented in TarBase 6.0 is 10,574 composed from 137 miRNAs and 5,707 mRNAs. Expression matrices for GBM consist of 144 miRNAs × 425 patients and 6,700 target genes × 425 patients. Among all possible 964,800 miRNA-mRNA pairs, the number of validated interactions is 9,073 from 144 miRNAs and 6,700 mRNAs.

### Survival analysis of miRNA and target mRNA

**Figure 1:**
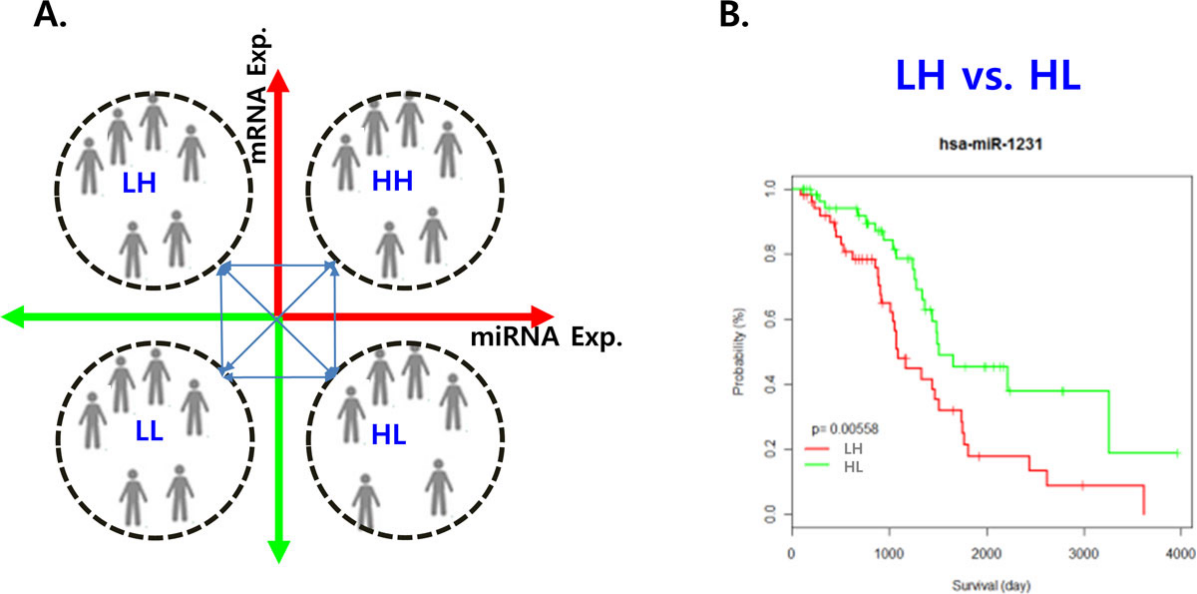
**Scheme of survival analysis with miRNA and mRNA**. Scheme of survival analysis with miRNA and mRNA A. The grouping method according to miRNA and mRNA expression profiles for survival analysis. The survival analysis based on the combination of expression levels of miRNA and mRNA. B. The survival curve between LH group and HL group.

We performed the survival analysis for patient groups using the Kaplan-Meier estimator. Each group was constructed based on the expression profiles of a miRNA-mRNA pair. There are four possible combinations of expression profiles (Figure 1(A)). One is a group with low expression of a miRNA and high expression of a mRNA. We defined this group as LH. On the other hand, a second group is HL that is high expression of miRNA and low expression of mRNA. The other groups, HH and LL, denote both high and both low expression of miRNA and mRNA, respectively.

We carried out the survival analysis of each miRNA-mRNA pair for six possible pairwise sets (HL:LH, HH:LL, HL:LH, HH:HL, LL:LH and LL:HL). Among these sets, our focus was on the survival test between LH and HL (Figure 1(B)) as they are inversely related to the level of expression. The group HL indicates that high expression of miRNA causes low expression of mRNA through the mechanism that a miRNA represses or cleaves its target mRNA, while the group LH states that the low expression of miRNA results in the high expression of mRNA.

## Results

We investigated if the comparison between the two groups, HL:LH, showed any distinguishing characteristics compared to the other relationships between pairs in survival analysis. Thus, we performed the survival analysis of each miRNA-mRNA for six possible pairwise sets. Here, HH, HL, LH or LL indicates a patient group classified according to low or high expression level of a miRNA and mRNA. For example, HL means a patient group with high expression of a miRNA and low expression of mRNA. We performed the log-rank test of survival scores for all possible miRNA-mRNA pairs and then extracted the validated pairs whose interactions were shown in TarBase6.0. Table 1 shows the number of significant pairs with p-value of log-rank test <0.01 for both total pairs and validated pairs in each pairwise set. As the table indicates, there was not a substantial difference between the results. For example, in ovarian cancer, although the number of significant validated pairs in HL:LH is 261, which is higher than other pairs, the background number, 22,575 is higher as well. As a result, the number of pairs associated with survival in HL:LH might be less than we expected.

**Table 1:** Comparison of the number of significant pairs.

	Num of Significant Pairs (p-value < 0.01)	HH:LL	HL:LH	HH:HL	HH:LH	LL:LH	LL:HL
Ovarian Cancer	Validation Pair	257	261	113	200	143	248
	
	Total Pair	22,685	22,575	9,408	18,739	10,299	21,332

GBM	Validation Pair	234	250	229	171	161	229
	
	Total Pair	47,706	51,217	22,180	36,023	22,835	42,735

Comparison of the number of significant pairs (p-value <0.01) in total and validation for six pairwise survival test sets.

**Figure 2:**
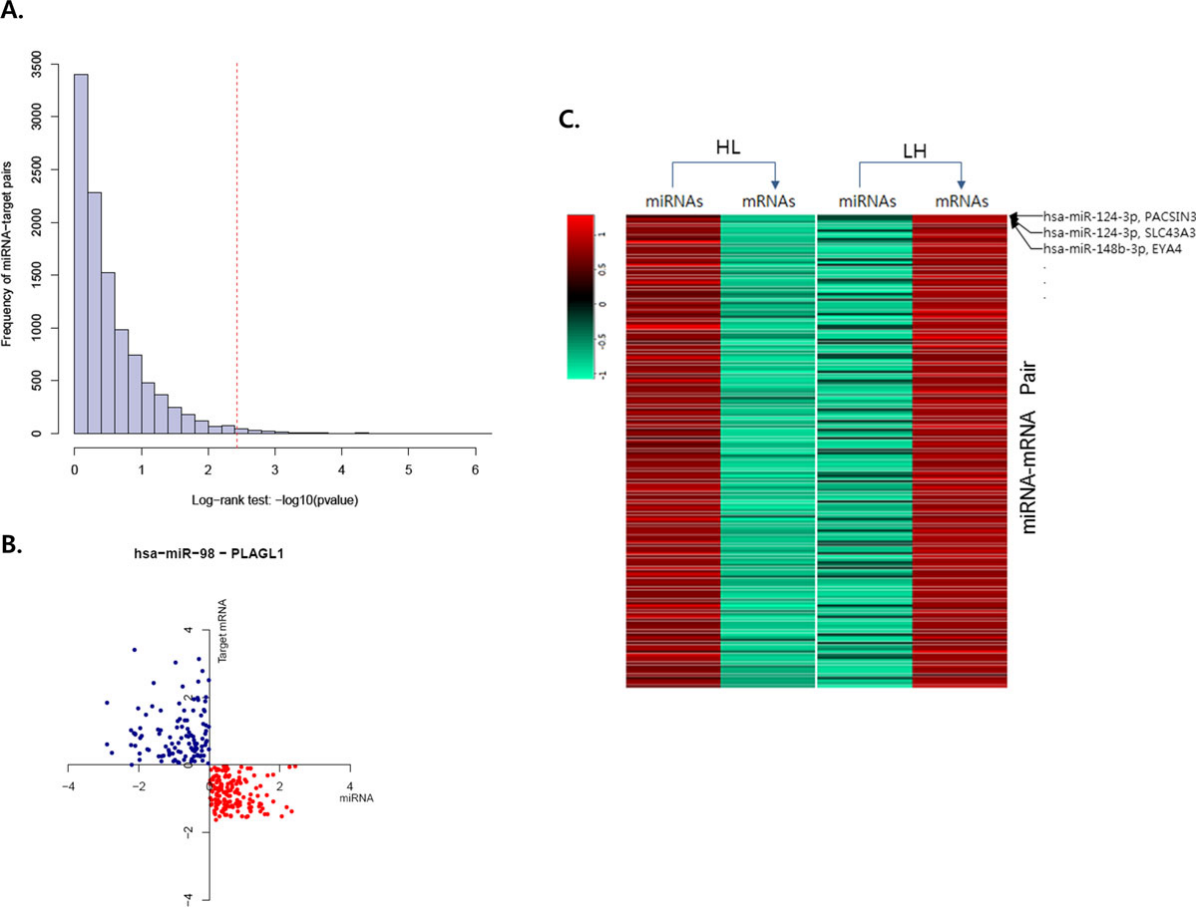
**Selecting significant miRNA-mRNA pairs from HL:LH (high miRNA & low mRNA expression group vs**. low miRNA & high mRNA expression group) type. Selecting significant miRNA-mRNA pairs from HL:LH (high miRNA & low mRNA expression group vs. low miRNA & high mRNA expression group) type. A. Distribution of significances by Log-rank test with HL:LH type in ovarian cancer. The dashed line indicates the threshold that the false discovery ratio is 0.01. B. The scatter plot of expression values for miR-98 and PLAGL1 pair among miRNA and mRNA pairs. Each point indicates a patient with HL:LH expression pattern. C. The heatmap of average expression values of miRNA and mRNAs for all significant pairs. Each cell indicates average expression value of a miRNA or mRNA in a group (i.e. HL or LH)

Therefore, we extracted miRNA-mRNA interaction pairs that showed significant difference between the HL group and LH group in survival rate using more stringent criteria. In Figure 2(A), we plotted the distribution of p-values corresponding to survival tests for 10,574 validated pairs in the ovarian cancer dataset. Each test was conducted from two groups containing different patients based on the expression values of miRNA and mRNA (Figure 2(B)). We obtained the threshold (corresponding to p-value < 3.66e^-3^) by calculating the false discovery rate (0.01) from a null model with a total of 776,016 pairs including most non-target relations. A total of 163 significant pairs with validated interactions were selected and they contained 22 miRNAs and 156 mRNAs. Expression patterns of all pairs are shown in Figure 2(C). The list of all significant interacting pairs for ovarian cancer is described in Additional file 1 Supplemental Table 1 and the list for GBM is also shown in Additional file 1 Supplemental Table 2. The survival difference between HL group and LH group suggests that the selected miRNA and its target mRNA might affect the progression of cancer in a coordinated fashion.

**Table 2:** Top 20 ranked miRNA-mRNA interaction pairs (OV).

miRNA	Gene	p-value (HL:LH)	Gene Description
hsa-miR-124-3p	*PACSIN3*	4.02E-05	Protein Kinase C And Casein Kinase Substrate In Neurons Protein

hsa-miR-124-3p	*SLC43A3*	5.19E-05	Solute carrier family 43, member 3

hsa-miR-148b-3p	*EYA4*	5.45E-05	Eyes Absent (Drosophila) Homolog 4

hsa-miR-148a-3p	*GAS1*	7.66E-05	Growth arrest-specific 1

hsa-miR-374b-5p	*TAF7*	1.38E-04	TAF7 RNA Polymerase II, TATA Box Binding Protein (TBP)-Associated Factor

hsa-miR-124-3p	*SDF2L1*	1.63E-04	Stromal Cell Derived Factor 2 Like Protein 1

hsa-miR-98	*SLC35D2*	1.83E-04	Solute carrier family 35, member D2

hsa-miR-98	PLAGL1*	1.91E-04	Pleomorphic adenoma gene-like 1

hsa-miR-124-3p	*SERP1*	2.09E-04	Stress-associated endoplasmic reticulum protein 1

hsa-miR-196a-5p	*LGR4*	3.36E-04	Leucine-Rich Repeat Containing G Protein-Coupled Receptor 4

hsa-miR-148b-3p	*APBB2*	3.86E-04	Amyloid beta (A4) precursor protein-binding, family B, member 2

hsa-miR-98	*FNDC3A*	3.90E-04	Fibronectin type III domain containing 3A

hsa-miR-98	MTUS1*	4.03E-04	Microtubule associated tumor suppressor 1

hsa-miR-98	*CDKAL1*	4.47E-04	CDK5 regulatory subunit associated protein 1-like 1

hsa-miR-98	*ZBED4*	4.59E-04	Zinc finger, BED-type containing 4

hsa-miR-124-3p	*FCHSD2*	5.28E-04	FCH and double SH3 domains 2

hsa-miR-98	*UST*	5.69E-04	Uronyl-2-Sulfotransferase

hsa-miR-124-3p	ELF4*	6.22E-04	E74-Like Factor 4 (Ets Domain Transcription Factor)

hsa-miR-98	*GNA12*	6.52E-04	Guanine Nucleotide Binding Protein (G Protein) Alpha 12

hsa-miR-98	IFNGR1*	6.69E-04	Interferon gamma receptor 1

Top 20 ranked miRNA-mRNA interaction pairs associated with survival difference from the ovarian cancer dataset.

* The functional roles in ovarian cancer have been known.

Among top 20 ranked miRNA-mRNA interaction pairs, the roles of four targets including PLAGL1, MTUS1, MEF and IFNGR1 have been reported in ovarian cancer (Table 2). miR-148a was down-regulated in ovarian cancer cell lines and might be involved in the carcinogenesis of ovarian cancer [21,22]. Previous research reported that overexpression of miR-148b in ovarian cancer tissues was not associated with any of the pathological features of patients with ovarian cancer. It suggested that miR-148b might be involved in the early stage of ovarian carcinogenesis [23]. Although other miRNAs including miR-196a, miR-374b, miR-124, and miR-98 have not been reported for associations with ovarian cancer, they are related with the oncogenic phenotype or their expression of other cancers [24-27].

Several miRNAs are dominated in significant miRNA-mRNA pairs. miR-98 and miR-148b-3p have more than 446 and 187 target genes, respectively. miR-98 or miR-148b-3p itself shows a significant survival difference between high and low expression (pvalue < 0.0077 and pvalue < 0.0014), while miR-124-3p with 979 targets genes shows a borderline significance (pvalue < 0.0051).

LOT1(PLAGL1/ZAC1) is known to possess anti-proliferative effects and is frequently silenced in ovarian cancer and breast cancer [28]. Previous studies suggest that a shortage of the PLAGL1 protein might impair its role in regulating the cell cycle and interfere with apoptosis. Consequently, cells may grow and divide too quickly in an uncontrolled manner. Mitochondrial tumor suppressor 1 (MTUS1) is a newly identified candidate tumor suppressor gene [29]. Previous studies have shown that MTUS1 expression levels are down-regulated in cancers of the colon, ovary, pancreas, head and neck, and breast cancer [30]. MEF (myeloid ELF1-like factor, also known as ELF4) is expressed in a significant proportion of ovarian carcinomas [31]. The oncogenic activity of MEF was shown by the ability of MEF to transform NIH3T3 cells, and induce the formation of tumors in nude mice. The expression level of IFNGR1 in a typical ovarian cancer population varies, with 22% of them displaying a complete loss of the IFNγ receptor [32]. Low levels of receptor expression seem to have a negative effect on survival and are unrelated to other pathologic variables. Therefore, low expression of IFNGR1 could be regarded as an independent prognostic marker in ovarian cancer. Although we have found only several previously reported functional roles related to ovarian cancer, they hint at the possibility that other target genes might be associated with ovarian cancer development and progression.

From the analysis of GBM dataset, we could find functional evidences of four targets including CDKN1A, MTAP, KIT and ATM among top 20 ranked miRNA-mRNA interaction pairs have been reported in GBM cancer (Table 3). The roles of most top-ranked miRNAs in GBM have been reported in previous literatures. miR-34a is a transcriptional target of p53 and it also suppresses brain tumor growth by targeting c-Met and Notch[33]. The significant correlation between miR-106a expression levels and overall survival was observed in a large set of FFPE GBM specimen and it could be used as an independent prognostic biomarker in GBM patients[34]. Previous study has reported that miR-221/222 cluster is involved in regulation of cell cycle progression and cell proliferation by targeting p27 and p57 [35]. Real-time PCR experiment revealed that the expression level of miR-26b was inversely correlated with the grade of glioma[36]. Thus it suggests that miR-26b may act as a tumor suppressor in GBM. miR-17 expression was higher in glioma tissue and it is significantly related to poor overall survival[37].

**Table 3:** Top 20 ranked miRNA-mRNA interaction pairs (GBM).

miRNA	Gene	p-value (HL:LH)	Gene Description
hsa-miR-19b-3p	*GPATCH8*	8.37E-06	G Patch Domain Containing 8

hsa-miR-19b-3p	*CLIP1*	2.01E-05	CAP-GLY domain containing linker protein 1

hsa-miR-19b-3p	*CTR9*	2.21E-05	Ctr9, Paf1/RNA polymerase II complex component, homolog (S. cerevisiae)

hsa-miR-34a-5p	*HIST3H2A*	3.11E-05	Histone cluster 3, H2a

hsa-miR-106a-5p	CDKN1A*	4.43E-05	Cyclin-dependent kinase inhibitor 1A (p21, Cip1)

hsa-miR-145-5p	FAM3C*	6.87E-05	Family with sequence similarity 3, member C

hsa-miR-19b-3p	*PRKAA1*	8.53E-05	Protein kinase, AMP-activated, alpha 1 catalytic subunit

hsa-miR-34a-5p	MTAP*	8.94E-05	Methylthioadenosine phosphorylase

hsa-miR-20a-5p	CDKN1A*	9.77E-05	Cyclin-dependent kinase inhibitor 1A (p21, Cip1)

hsa-miR-148a-3p	*HOXC8*	1.20E-04	Homeobox C8

hsa-miR-221-3p	*TFAP2A*	1.28E-04	Transcription factor AP-2 alpha (activating enhancer binding protein 2 alpha)

hsa-miR-19a-3p	*HOXA5*	1.29E-04	Homeobox A5

hsa-miR-200b-3p	*RIN2*	1.37E-04	Ras and Rab interactor 2

hsa-miR-26b-5p	*CLEC5A*	1.43E-04	C-type lectin domain family 5, member A

hsa-miR-19b-3p	*PSMD9*	1.77E-04	Proteasome (prosome, macropain) 26S subunit, non-ATPase, 9

hsa-miR-19b-3p	*LPHN2*	1.91E-04	Latrophilin 2

hsa-miR-34a-5p	*ATXN2L*	2.08E-04	Ataxin 2-like

hsa-miR-17-5p	CDKN1A*	2.44E-04	Cyclin-dependent kinase inhibitor 1A (p21, Cip1)

hsa-miR-222-3p	KIT*	2.50E-04	V-kit Hardy-Zuckerman 4 feline sarcoma viral oncogene homolog

hsa-miR-19b-3p	ATM*	2.54E-04	Ataxia telangiectasia mutated

Top 20 ranked miRNA-mRNA interaction pairs associated with survival difference from the GBM dataset.

* The functional roles in GBM cancer have been known.

Previous quantitative real-time PCR revealed overexpression of CDKN1A in primary GBM[38]. This result suggests that CDKN1A expression is regarded as a putative marker to distinguish primary GBM from secondary GBM. The gene for methylthioadenosine phosphorylase (MTAP) is located closely to the gene CDKN2A. MTAP-deficiency in many tumors that have been most resistant to treatment occurs commonly. Especially 70% of glioblastoma lack MTAP. Amplification of KIT in 17 (4.4%) glioblastomas was reveled from screening of 390 glioblastomas[40]. A borderline positive association (p=0.0579) between KIT amplification and TP53 mutation was also observed. ATM expression is correlated with radioresistance in primary GBM cells in culture. Genes encoding components of the DNA-damage response (DDR) pathway are frequently altered in human GBM patients and the ATM/Chk2/p53 cascade suppresses GBM formation[41].

**Figure 3:**
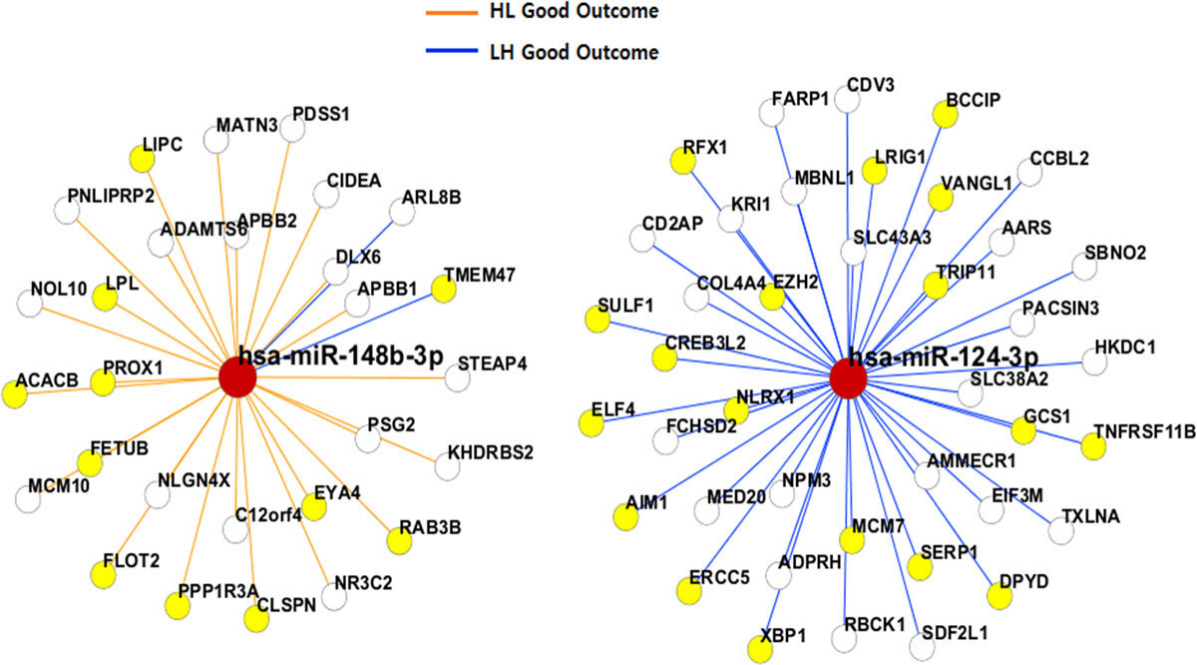
**Network visualization of miRNA-mRNA interaction pairs in terms of clinical outcome in ovarian cancer patients**. Network visualization of miRNA-mRNA interaction pairs in terms of clinical outcome in ovarian cancer patients. Highlighted nodes indicate genes that are associated with Neoplasm/Cancer-related condition.

Figure 3 displays the network of miRNA-target mRNA interactions that are associated with the difference of clinical outcome of OV cancer patients. It shows that many target genes in the network, indicated by highlighted nodes, are associated with neoplasm or cancer-related conditions. Some are interaction pairs where HL groups show good outcomes while others are when LH groups have good outcome. This network implies that the increased expression of miR-148b is significantly associated with survival rate for ovarian cancer, which agrees with the previous observation [23]. In contrast, the low level of expression of miR-124 is shown to have strong association with survival rate. The complete network of interactions can be found in Additional file 1 Supplementary Figure 1.

## Conclusions

We have introduced the integrated analysis of survival test with datasets of miRNA and mRNA expression profiles, as well as clinical information in ovarian cancer and GBM. We have seen that the combined expression patterns between miRNAs and mRNAs can distinguish between risk groups related to co-regulation of both. In addition, we have presented supporting evidence for functional roles of miRNA and their targets in specific cancer from the literature. Our approach can be utilized to detect clinical and therapeutic miRNAs and their targets related to outcome of several cancers.

## Competing interests

The authors declare that they have no competing interests.

## Authors' contributions

JGJ proposed the idea, organized overall procedure and carried out the analysis. DK built the dataset of computational experiments and developed the concept. SYL interpreted the results. HJK improved the manuscript writing and reviewed the manuscript. JHK provided intellectual guidance and mentorship.

## Supplementary Material

Additional file 1 **Survival analysis of six different test set and network of significant miRNA-mRNA interaction pairs**. Supplemental Table 1. Survival analysis of six different test sets or validated miRNA-mRNA interactions with ovarian cancer dataset. Supplemental able 2. Survival analysis of six different test sets for validated miRNA-mRNA interactions with GBM dataset. Supplemental Figure 1. Network visualization of all significant iRNA-mRNA interaction pairs in terms of clinical outcome in ovarian cancer patients. Click here for file

## Declarations

Funding for open access charge: National Research Foundation of Korea (NRF).

This article has been published as part of *BMC Medical Genomics *Volume 7 Supplement 1, 2014: Selected articles from the 3rd Translational Bioinformatics Conference (TBC/ISCB-Asia 2013). The full contents of the supplement are available online at http://www.biomedcentral.com/bmcmedgenomics/supplements/7/S1.
