# Supplementary material for: Integrated analysis of microRNA-target interactions with clinical outcomes for cancers
Source: BMC Med Genomics. 2014 May 8;7(Suppl 1):S10. doi: 10.1186/1755-8794-7-S1-S10 (PMC4101396; doi:10.1186/1755-8794-7-S1-S10)
Supplement: Additional file 1 — Survival analysis of six different test set and network of significant miRNA-mRNA interaction pairs. Supplemental Table 1. Survival analysis of six different test sets or validated miRNA-mRNA interactions with ovarian cancer dataset. Supplemental able 2. Survival analysis of six different test sets for validated miRNA-mRNA interactions with GBM dataset. Supplemental Figure 1. Network visualization of all significant iRNA-mRNA interaction pairs in terms of clinical outcome in ovarian cancer patients. [file 1755-8794-7-S1-S10-S1.pdf]

Supplemental Table 1. Survival analysis of six different test sets for validated miRNA-mRNA interactions with ovarian cancer dataset.

Num	miRNA	Gene	p-value (HH:LL)	p-value (HL:LH)	p-value (HH:HL)	p-value (HH:LH)	p-value (LL:LH)	p-value (LL:HL)
1	**hsa-miR-124-3p**	**PACSIN3**	1.97E-01	4.02E-05	3.24E-02	2.73E-01	6.74E-04	1.02E-01
2	**hsa-miR-124-3p**	**SLC43A3**	2.56E-01	5.19E-05	1.36E-03	4.18E-01	3.40E-01	3.82E-03
3	**hsa-miR-148b-3p**	**EYA4**	4.40E-01	5.45E-05	7.55E-01	1.41E-03	3.68E-03	1.71E-01
4	**hsa-miR-148a-3p**	**GAS1**	5.22E-01	7.66E-05	1.07E-03	8.52E-01	7.49E-01	1.76E-04
5	**hsa-miR-374b-5p**	**TAF7**	6.78E-01	1.38E-04	9.21E-01	8.32E-05	1.83E-03	5.96E-01
6	**hsa-miR-124-3p**	**SDF2L1**	3.32E-01	1.63E-04	2.48E-02	7.32E-01	4.22E-02	4.08E-02
7	**hsa-miR-98**	**SLC35D2**	5.33E-01	1.83E-04	6.44E-01	2.20E-03	7.10E-03	3.83E-01
8	**hsa-miR-98**	**PLAGL1**	8.92E-01	1.91E-04	2.05E-01	2.90E-02	2.85E-02	1.61E-01
9	**hsa-miR-124-3p**	**SERP1**	4.36E-01	2.09E-04	3.39E-02	5.29E-01	2.09E-02	6.35E-02
10	**hsa-miR-196a-5p**	**LGR4**	2.95E-02	3.36E-04	2.98E-04	2.68E-01	1.52E-01	1.62E-02
11	**hsa-miR-148b-3p**	**APBB2**	8.06E-01	3.86E-04	2.79E-02	2.98E-01	2.14E-01	1.36E-02
12	**hsa-miR-98**	**FNDC3A**	6.40E-01	3.90E-04	6.99E-01	8.01E-04	1.57E-03	8.52E-01
13	**hsa-miR-98**	**MTUS1**	5.33E-01	4.03E-04	9.62E-01	1.63E-03	8.34E-03	5.67E-01
14	**hsa-miR-98**	**CDKAL1**	5.67E-01	4.47E-04	5.00E-01	2.18E-02	3.61E-02	1.92E-01
15	**hsa-miR-98**	**ZBED4**	4.00E-01	4.59E-04	8.87E-01	8.52E-04	5.81E-03	5.15E-01
16	**hsa-miR-124-3p**	**FCHSD2**	9.18E-01	5.28E-04	7.86E-04	4.76E-01	4.07E-01	1.24E-04
17	**hsa-miR-98**	**UST**	8.72E-01	5.69E-04	8.75E-02	9.66E-02	1.08E-01	4.48E-02
18	**hsa-miR-124-3p**	**ELF4**	7.15E-01	6.22E-04	2.75E-02	6.69E-01	1.83E-01	1.84E-02
19	**hsa-miR-98**	**GNA12**	6.25E-01	6.52E-04	2.93E-01	7.21E-02	7.93E-02	1.27E-01
20	**hsa-miR-98**	**IFNGR1**	5.37E-01	6.69E-04	6.46E-01	5.34E-04	4.15E-03	6.47E-01
21	**hsa-miR-98**	**CPA4**	3.12E-01	6.76E-04	7.55E-01	9.38E-03	5.86E-02	1.44E-01
22	**hsa-miR-148a-3p**	**MTMR9**	1.72E-01	6.78E-04	7.52E-01	1.58E-02	1.51E-01	6.36E-02
23	**hsa-miR-124-3p**	**AARS**	9.34E-01	6.97E-04	2.80E-02	7.18E-01	4.02E-01	1.11E-02
24	**hsa-miR-1**	**PACSIN3**	2.39E-02	7.34E-04	3.01E-02	5.21E-01	6.89E-04	7.95E-01
25	**hsa-miR-98**	**CAP1**	4.10E-01	7.45E-04	3.94E-01	3.02E-02	1.45E-01	7.77E-02
26	**hsa-miR-98**	**CHST3**	6.33E-01	7.68E-04	3.75E-01	4.69E-02	7.28E-02	1.35E-01
27	**hsa-miR-98**	**RASAL2**	4.18E-01	8.29E-04	7.43E-01	1.10E-02	4.49E-02	2.97E-01
28	**hsa-miR-124-3p**	**HKDC1**	7.44E-01	8.36E-04	9.99E-04	4.06E-01	5.74E-01	2.55E-04
29	**hsa-miR-98**	**PRKAA2**	7.64E-01	8.56E-04	1.95E-01	3.69E-02	1.10E-01	6.90E-02
30	**hsa-miR-124-3p**	**EZH2**	7.28E-01	8.77E-04	3.67E-02	4.74E-01	1.06E-01	1.97E-02
31	**hsa-miR-148b-3p**	**LIPC**	9.38E-01	9.00E-04	3.91E-02	3.97E-01	1.93E-01	7.31E-03
32	**hsa-miR-24-3p**	**PACSIN3**	1.52E-02	9.30E-04	9.62E-03	3.93E-01	1.24E-03	6.73E-01
33	**hsa-miR-148b-3p**	**PSG2**	4.68E-01	9.59E-04	2.60E-01	3.37E-02	1.78E-01	1.21E-01
34	**hsa-miR-148b-3p**	**LPL**	7.29E-01	1.02E-03	4.45E-01	6.79E-02	3.10E-01	6.18E-02
35	**hsa-miR-124-3p**	**RFX1**	6.17E-01	1.02E-03	1.79E-01	7.68E-02	8.68E-04	4.00E-01
36	**hsa-miR-21-5p**	**CPS1**	1.91E-02	1.03E-03	1.59E-02	2.80E-01	6.30E-04	9.72E-01
37	**hsa-miR-148a-3p**	**TMEM9B**	2.69E-01	1.04E-03	2.53E-01	3.95E-02	4.27E-01	1.26E-02
38	**hsa-miR-98**	**YOD1**	6.87E-01	1.06E-03	1.19E-01	9.42E-02	2.51E-01	6.11E-02
39	**hsa-miR-124-3p**	**MBNL1**	6.40E-01	1.09E-03	4.60E-03	8.65E-01	6.28E-01	1.22E-03
40	**hsa-miR-124-3p**	**TXLNA**	9.39E-01	1.10E-03	3.56E-02	4.28E-01	1.45E-01	2.25E-02
41	**hsa-miR-374b-5p**	**C1orf56**	5.03E-01	1.11E-03	1.27E-01	7.89E-02	2.38E-01	1.66E-02
42	**hsa-miR-98**	**ARSJ**	2.12E-01	1.13E-03	8.18E-01	5.79E-03	4.56E-02	2.10E-01
43	**hsa-miR-98**	**SCG2**	5.76E-01	1.14E-03	2.78E-01	7.65E-02	1.06E-01	4.90E-02
44	**hsa-miR-124-3p**	**ERCC5**	8.32E-01	1.18E-03	3.84E-03	6.57E-01	5.34E-01	5.76E-04
45	**hsa-miR-124-3p**	**CDV3**	6.99E-01	1.25E-03	1.05E-02	8.58E-01	7.22E-01	2.57E-03
46	**hsa-miR-148b-3p**	**NLGN4X**	4.66E-01	1.31E-03	4.60E-01	2.96E-02	1.21E-01	1.43E-01
47	**hsa-miR-98**	**TEAD1**	5.79E-01	1.33E-03	1.18E-01	1.08E-01	3.48E-01	3.14E-02
48	**hsa-miR-98**	**MITF**	5.33E-01	1.37E-03	2.54E-01	3.65E-02	8.93E-02	8.53E-02
49	**hsa-miR-124-3p**	**CCBL2**	7.85E-01	1.42E-03	6.17E-02	5.92E-01	1.49E-01	6.50E-02
50	**hsa-miR-124-3p**	**CREB3L2**	9.57E-01	1.45E-03	1.31E-02	5.34E-01	3.89E-01	3.42E-03
51	**hsa-miR-98**	**RND3**	6.16E-01	1.50E-03	5.22E-01	1.84E-02	8.26E-02	2.12E-01
52	**hsa-miR-148b-3p**	**NR3C2**	2.61E-01	1.51E-03	8.44E-01	2.09E-03	1.35E-02	3.38E-01
53	**hsa-miR-98**	**SNX24**	7.01E-01	1.54E-03	4.51E-02	2.31E-01	4.88E-01	1.01E-02
54	**hsa-miR-98**	**PDSS1**	3.96E-01	1.57E-03	7.18E-01	3.10E-04	8.90E-03	5.62E-01
55	**hsa-miR-124-3p**	**XBP1**	5.47E-01	1.64E-03	6.12E-02	7.52E-01	1.38E-01	6.18E-02
56	**hsa-miR-16-5p**	**PACSIN3**	5.52E-03	1.75E-03	2.13E-02	4.64E-01	5.47E-04	5.00E-01
57	**hsa-miR-374b-5p**	**MINPP1**	2.67E-01	1.84E-03	3.10E-01	3.50E-02	2.12E-01	6.16E-02
58	**hsa-miR-124-3p**	**AIM1**	9.04E-01	1.86E-03	6.09E-02	8.26E-01	4.42E-01	2.05E-02
59	**hsa-miR-373-3p**	**CD83**	7.50E-01	1.86E-03	4.66E-02	3.07E-01	3.29E-01	7.19E-03
60	**hsa-miR-98**	**CDKN1B**	5.30E-01	1.87E-03	5.54E-01	1.47E-02	7.35E-02	2.21E-01

61	**hsa-miR-148b-3p**	**PROX1**	5.49E-01	1.94E-03	5.63E-02	3.01E-01	5.45E-01	3.32E-03
62	**hsa-miR-98**	**RGS16**	3.47E-01	2.00E-03	9.09E-01	1.26E-02	6.99E-02	2.31E-01
63	**hsa-miR-148b-3p**	**DLX6**	3.50E-01	2.01E-03	2.17E-01	6.04E-02	2.75E-01	3.38E-02
64	**hsa-miR-98**	**MAGEA12**	7.66E-01	2.02E-03	1.34E-01	2.64E-01	3.07E-01	2.40E-02
65	**hsa-miR-148b-3p**	**KHDRBS2**	4.82E-01	2.06E-03	1.05E-02	5.77E-01	9.92E-01	1.49E-03
66	**hsa-miR-374b-5p**	**NRXN3**	5.52E-01	2.08E-03	7.93E-02	3.10E-01	6.72E-01	1.90E-02
67	**hsa-miR-148a-3p**	**GNB5**	1.40E-01	2.10E-03	4.87E-01	3.35E-02	4.01E-01	1.57E-02
68	**hsa-miR-124-3p**	**NLRX1**	9.45E-01	2.13E-03	1.39E-01	3.62E-01	1.10E-01	8.99E-02
69	**hsa-miR-30a-5p**	**SLC33A1**	3.49E-01	2.19E-03	7.99E-02	2.30E-01	2.59E-02	2.93E-01
70	**hsa-miR-98**	**HSPB7**	5.01E-01	2.21E-03	2.99E-01	1.01E-01	3.12E-01	5.78E-02
71	**hsa-miR-98**	**GATA6**	4.59E-01	2.26E-03	6.03E-02	1.87E-01	5.32E-01	1.57E-02
72	**hsa-miR-124-3p**	**MED20**	9.67E-01	2.26E-03	5.94E-01	8.22E-02	3.00E-03	5.18E-01
73	**hsa-miR-148b-3p**	**FETUB**	4.98E-01	2.26E-03	2.18E-01	9.11E-02	2.54E-01	2.68E-02
74	**hsa-miR-30b-5p**	**GFPT2**	6.20E-01	2.27E-03	3.46E-01	5.65E-02	8.96E-03	6.75E-01
75	**hsa-miR-124-3p**	**FARP1**	8.18E-01	2.29E-03	1.06E-01	3.87E-01	2.50E-01	4.65E-02
76	**hsa-miR-24-3p**	**ALDH5A1**	6.90E-02	2.31E-03	1.31E-01	1.98E-01	2.05E-03	8.16E-01
77	**hsa-miR-98**	**PDHB**	4.30E-01	2.34E-03	2.36E-01	6.61E-02	3.38E-01	6.00E-02
78	**hsa-miR-124-3p**	**MCM7**	9.48E-01	2.34E-03	1.14E-01	5.00E-01	1.61E-01	5.51E-02
79	**hsa-miR-148a-3p**	**GPATCH8**	1.43E-01	2.39E-03	8.93E-01	6.75E-03	1.85E-01	1.25E-01
80	**hsa-miR-26b-5p**	**NCAM2**	1.00E-01	2.40E-03	1.26E-01	9.54E-02	2.29E-03	9.37E-01
81	**hsa-miR-98**	**C10orf88**	2.65E-01	2.41E-03	4.58E-01	3.26E-02	2.22E-01	1.23E-01
82	**hsa-miR-124-3p**	**LRIG1**	8.56E-01	2.43E-03	1.15E-01	3.14E-01	2.39E-01	1.16E-01
83	**hsa-miR-124-3p**	**VANGL1**	9.02E-01	2.46E-03	1.78E-01	4.00E-01	8.33E-02	1.27E-01
84	**hsa-miR-148b-3p**	**CIDEA**	1.48E-01	2.47E-03	3.63E-01	2.86E-04	5.74E-03	5.12E-01
85	**hsa-miR-98**	**ADCY9**	4.52E-01	2.48E-03	4.87E-01	2.67E-02	1.32E-01	1.23E-01
86	**hsa-miR-98**	**COG5**	5.37E-01	2.49E-03	3.26E-01	5.05E-02	2.59E-01	1.10E-01
87	**hsa-miR-98**	**ZFAND5**	4.17E-01	2.51E-03	6.49E-01	8.23E-04	1.42E-02	6.63E-01
88	**hsa-miR-98**	**KLF10**	5.26E-01	2.55E-03	7.48E-01	7.65E-03	6.61E-02	3.44E-01
89	**hsa-miR-98**	**PXDN**	6.40E-01	2.57E-03	3.86E-02	2.55E-01	6.35E-01	1.13E-02
90	**hsa-miR-122-5p**	**FSTL3**	9.04E-01	2.60E-03	7.15E-01	1.69E-02	2.41E-03	7.37E-01
91	**hsa-miR-124-3p**	**SLC38A2**	9.78E-01	2.63E-03	1.50E-02	7.95E-01	7.19E-01	2.56E-03
92	**hsa-miR-148b-3p**	**ACACB**	5.03E-01	2.65E-03	1.58E-01	1.94E-01	6.11E-01	1.50E-02
93	**hsa-miR-124-3p**	**NPM3**	8.58E-01	2.66E-03	4.14E-02	7.81E-01	4.93E-01	1.39E-02
94	**hsa-miR-30a-5p**	**PDCD10**	5.85E-01	2.70E-03	3.19E-02	5.18E-01	1.93E-01	8.60E-02
95	**hsa-miR-421**	**PNMA2**	6.91E-01	2.71E-03	1.93E-01	2.51E-01	5.43E-02	2.35E-01
96	**hsa-miR-1**	**TDP1**	1.39E-01	2.77E-03	1.41E-02	9.26E-01	6.00E-02	1.32E-01
97	**hsa-miR-374b-5p**	**GABARAPL3**	4.20E-01	2.81E-03	1.41E-01	8.38E-02	5.08E-01	2.20E-02
98	**hsa-miR-340-5p**	**NET1**	2.82E-01	2.82E-03	8.65E-02	2.53E-01	1.48E-02	5.78E-01
99	**hsa-miR-98**	**PTTG1IP**	3.95E-01	2.83E-03	4.85E-01	2.94E-02	2.30E-01	1.10E-01
100	**hsa-miR-124-3p**	**RBCK1**	9.44E-01	2.86E-03	2.62E-02	8.96E-01	6.64E-01	4.44E-03
101	**hsa-miR-374b-5p**	**ADD3**	2.66E-01	2.97E-03	2.67E-02	3.94E-01	8.08E-01	1.77E-03
102	**hsa-miR-30a-5p**	**PAICS**	7.45E-01	3.03E-03	1.54E-01	1.78E-01	7.88E-02	2.90E-01
103	**hsa-miR-148a-3p**	**ADARB1**	1.48E-01	3.04E-03	2.84E-01	7.92E-02	7.07E-01	6.87E-03
104	**hsa-miR-98**	**NFIL3**	3.49E-01	3.10E-03	9.49E-01	3.16E-03	4.36E-02	4.21E-01
105	**hsa-miR-148b-3p**	**RAB3B**	5.90E-01	3.12E-03	9.15E-02	1.97E-01	3.38E-01	1.35E-02
106	**hsa-miR-124-3p**	**SULF1**	8.55E-01	3.16E-03	2.74E-02	8.45E-01	8.77E-01	6.64E-03
107	**hsa-miR-148b-3p**	**APBB1**	5.06E-01	3.28E-03	1.98E-01	1.38E-01	4.22E-01	3.63E-02
108	**hsa-miR-148a-3p**	**CYCS**	9.15E-02	3.41E-03	9.24E-01	1.22E-02	3.39E-01	4.15E-02
109	**hsa-miR-148b-3p**	**NOL10**	3.18E-01	3.42E-03	4.02E-01	4.91E-02	3.37E-01	5.01E-02
110	**hsa-miR-124-3p**	**TNFRSF11B**	8.81E-01	3.45E-03	2.00E-01	3.19E-01	9.74E-02	7.41E-02
111	**hsa-miR-148b-3p**	**ADAMTS6**	3.39E-01	3.52E-03	4.40E-01	2.89E-02	1.22E-01	6.90E-02
112	**hsa-miR-148b-3p**	**PPP1R3A**	2.20E-01	3.56E-03	9.43E-01	3.86E-03	6.81E-02	2.04E-01
113	**hsa-miR-98**	**TRAF3IP2**	5.03E-01	3.59E-03	4.61E-01	1.79E-02	1.48E-01	1.19E-01
114	**hsa-miR-124-3p**	**TRIP11**	5.50E-01	3.60E-03	9.13E-03	6.59E-01	8.61E-01	2.03E-03
115	**hsa-miR-124-3p**	**KRI1**	7.08E-01	3.63E-03	3.73E-02	9.88E-01	4.30E-01	9.98E-03
116	**hsa-miR-148a-3p**	**UQCRQ**	1.15E-01	3.64E-03	4.16E-01	3.25E-02	5.17E-01	1.26E-02
117	**hsa-miR-148b-3p**	**MATN3**	4.31E-01	3.68E-03	5.09E-01	1.04E-01	2.79E-01	9.00E-02
118	**hsa-miR-124-3p**	**SBNO2**	7.29E-01	3.69E-03	4.61E-02	9.88E-01	5.05E-01	1.46E-02
119	**hsa-miR-98**	**NEK3**	3.43E-01	3.75E-03	7.85E-01	4.63E-02	1.78E-01	1.19E-01
120	**hsa-miR-148b-3p**	**PDSS1**	3.42E-01	3.78E-03	7.81E-01	2.50E-02	1.58E-01	1.09E-01
121	**hsa-miR-124-3p**	**AMMECR1**	9.75E-01	3.80E-03	2.99E-01	3.32E-01	6.80E-02	1.70E-01
122	**hsa-miR-122-5p**	**CHST3**	7.73E-01	3.85E-03	5.49E-01	4.99E-02	1.91E-02	3.20E-01
123	**hsa-miR-124-3p**	**GCS1**	8.12E-01	3.87E-03	2.90E-02	7.50E-01	4.61E-01	4.54E-03
124	**hsa-miR-98**	**TRIB1**	3.41E-01	3.94E-03	5.21E-01	3.29E-02	2.45E-01	8.61E-02
125	**hsa-miR-1**	**SERP1**	1.46E-01	3.96E-03	4.83E-02	6.61E-01	1.85E-02	4.05E-01

126	**hsa-miR-124-3p**	**COL4A4**	9.09E-01	3.97E-03	6.34E-01	8.03E-02	2.13E-02	2.38E-01
127	**hsa-miR-148b-3p**	**C12orf4**	3.40E-01	3.99E-03	3.31E-01	1.23E-01	3.73E-01	2.53E-02
128	**hsa-miR-148b-3p**	**PNLIPRP2**	4.44E-01	4.05E-03	2.48E-01	1.06E-01	2.28E-01	3.43E-02
129	**hsa-miR-98**	**SNAP23**	1.60E-01	4.10E-03	4.03E-01	1.27E-03	4.49E-02	4.66E-01
130	**hsa-miR-128**	**STK3**	2.14E-01	4.11E-03	3.06E-01	1.66E-01	5.24E-03	8.32E-01
131	**hsa-miR-98**	**RPAP1**	3.24E-01	4.13E-03	8.74E-01	3.61E-03	5.22E-02	2.91E-01
132	**hsa-miR-98**	**TMEM45A**	3.36E-01	4.16E-03	6.67E-01	2.90E-02	1.89E-01	1.69E-01
133	**hsa-miR-148b-3p**	**TMEM47**	3.99E-01	4.18E-03	2.32E-01	5.39E-02	4.32E-01	5.78E-02
134	**hsa-miR-148b-3p**	**FLOT2**	5.23E-01	4.20E-03	1.33E-01	2.47E-01	7.39E-01	8.89E-03
135	**hsa-miR-98**	**CDC23**	4.05E-01	4.24E-03	9.04E-01	2.83E-03	3.67E-02	3.52E-01
136	**hsa-miR-124-3p**	**BCCIP**	8.50E-01	4.29E-03	2.21E-02	8.96E-01	8.56E-01	3.13E-03
137	**hsa-miR-98**	**MYC**	3.68E-01	4.30E-03	1.53E-01	1.67E-01	6.48E-01	2.59E-02
138	**hsa-miR-148b-3p**	**STEAP4**	8.53E-01	4.33E-03	1.27E-02	9.63E-01	8.38E-01	6.00E-04
139	**hsa-miR-30a-5p**	**CD44**	8.38E-01	4.34E-03	3.70E-01	6.84E-02	2.93E-02	4.36E-01
140	**hsa-miR-124-3p**	**DPYD**	9.88E-01	4.36E-03	1.18E-01	2.71E-01	1.30E-01	4.14E-02
141	**hsa-miR-98**	**ZNF281**	4.52E-01	4.41E-03	5.25E-01	3.45E-02	1.39E-01	1.39E-01
142	**hsa-miR-98**	**EIF4E2**	2.90E-01	4.43E-03	2.18E-01	1.64E-01	6.66E-01	2.79E-02
143	**hsa-miR-98**	**TEAD4**	4.57E-01	4.45E-03	3.40E-02	3.78E-01	9.15E-01	6.48E-03
144	**hsa-miR-98**	**UBE2D3**	2.93E-01	4.51E-03	8.30E-01	4.65E-03	6.33E-02	4.50E-01
145	**hsa-miR-98**	**SERTAD2**	3.61E-01	4.53E-03	1.71E-01	1.60E-01	6.18E-01	2.52E-02
146	**hsa-miR-124-3p**	**EIF3M**	7.80E-01	4.57E-03	1.46E-02	9.91E-01	6.11E-01	1.74E-03
147	**hsa-miR-32-5p**	**GFPT2**	2.79E-01	4.59E-03	3.81E-01	1.03E-01	5.03E-03	8.36E-01
148	**hsa-miR-98**	**KCNJ15**	5.40E-01	4.63E-03	2.16E-01	1.73E-01	4.32E-01	1.91E-02
149	**hsa-miR-124-3p**	**CD2AP**	7.93E-01	4.67E-03	3.16E-01	3.57E-01	4.32E-02	1.99E-01
150	**hsa-miR-30a-5p**	**IFRD1**	5.88E-01	4.69E-03	1.74E-01	1.37E-01	4.86E-02	4.33E-01
151	**hsa-miR-138-5p**	**EZH2**	5.63E-01	4.73E-03	1.60E-01	3.29E-01	5.01E-02	2.96E-01
152	**hsa-miR-26b-5p**	**LSAMP**	1.47E-01	4.76E-03	1.82E-01	2.11E-01	6.10E-03	8.76E-01
153	**hsa-miR-122-5p**	**NPEPPS**	9.59E-01	4.77E-03	3.40E-01	9.56E-02	3.44E-02	3.38E-01
154	**hsa-miR-124-3p**	**ADPRH**	8.48E-01	4.77E-03	6.06E-02	6.61E-01	7.74E-01	2.29E-02
155	**hsa-miR-148b-3p**	**ARL8B**	2.95E-01	4.80E-03	6.20E-01	2.84E-02	1.93E-01	9.28E-02
156	**hsa-miR-98**	**KIAA0406**	3.03E-01	4.84E-03	7.35E-01	2.32E-03	3.24E-02	5.28E-01
157	**hsa-miR-374b-5p**	**PARD6B**	2.48E-01	4.86E-03	6.28E-01	2.56E-02	2.20E-01	6.85E-02
158	**hsa-miR-98**	**ZNF473**	4.20E-01	4.88E-03	1.43E-01	1.60E-01	5.26E-01	2.10E-02
159	**hsa-miR-98**	**ERO1L**	3.77E-01	4.90E-03	5.41E-01	2.81E-02	2.21E-01	7.70E-02
160	**hsa-miR-98**	**WNT10B**	3.42E-01	4.91E-03	2.83E-01	7.59E-02	4.28E-01	4.79E-02
161	**hsa-miR-559**	**ERBB2**	5.99E-01	4.91E-03	4.50E-01	7.31E-02	3.98E-02	1.13E-01
162	**hsa-miR-148b-3p**	**CLSPN**	2.13E-01	4.93E-03	6.07E-01	7.47E-02	4.45E-01	4.42E-02
163	**hsa-miR-148b-3p**	**MCM10**	3.14E-01	4.94E-03	5.64E-01	4.15E-02	1.98E-01	7.19E-02
164	**hsa-let-7d-5p**	**KPNA5**	9.87E-01	4.96E-03	5.90E-01	5.93E-02	3.76E-02	7.33E-01

Supplemental Table 2. Survival analysis of six different test sets for validated miRNA-mRNA interactions with GBM dataset.

Num	miRNA	Gene	p-value (HH:LL)	p-value (HL:LH)	p-value (HH:HL)	p-value (HH:LH)	p-value (LL:LH)	p-value (LL:HL)
1	**hsa-miR-19b-3p**	**GPATCH8**	5.32E-01	8.37E-06	5.40E-03	5.62E-02	1.96E-01	8.47E-04
2	**hsa-miR-19b-3p**	**CLIP1**	9.75E-01	2.01E-05	4.05E-02	5.40E-02	7.92E-02	1.77E-02
3	**hsa-miR-19b-3p**	**CTR9**	5.60E-01	2.21E-05	3.61E-02	3.72E-02	1.97E-01	1.41E-02
4	**hsa-miR-34a-5p**	**HIST3H2A**	2.91E-01	3.11E-05	1.64E-01	6.28E-03	2.17E-04	8.64E-01
5	**hsa-miR-106a-5p**	**CDKN1A**	5.07E-01	4.43E-05	7.10E-02	2.74E-02	1.61E-01	2.27E-02
6	**hsa-miR-145-5p**	**FAM3C**	1.05E-01	6.87E-05	2.00E-02	4.47E-02	2.07E-03	2.39E-01
7	**hsa-miR-19b-3p**	**PRKAA1**	4.91E-01	8.53E-05	2.69E-01	9.65E-03	1.59E-02	4.97E-02
8	**hsa-miR-34a-5p**	**MTAP**	5.03E-01	8.94E-05	3.53E-02	1.12E-01	3.01E-02	2.41E-01
9	**hsa-miR-20a-5p**	**CDKN1A**	4.54E-01	9.77E-05	1.23E-02	2.13E-01	5.75E-01	2.18E-03
10	**hsa-miR-148a-3p**	**HOXC8**	4.04E-01	1.20E-04	2.54E-01	2.17E-02	1.58E-01	1.93E-02
11	**hsa-miR-221-3p**	**TFAP2A**	5.86E-01	1.28E-04	2.75E-01	2.81E-02	1.18E-01	9.40E-02
12	**hsa-miR-19a-3p**	**HOXA5**	1.73E-01	1.29E-04	1.38E-01	3.34E-03	2.85E-01	6.39E-03
13	**hsa-miR-200b-3p**	**RIN2**	8.87E-01	1.37E-04	3.78E-02	2.67E-01	2.75E-01	2.57E-03
14	**hsa-miR-26b-5p**	**CLEC5A**	4.99E-03	1.43E-04	5.35E-04	5.38E-01	1.51E-03	5.55E-01
15	**hsa-miR-19b-3p**	**PSMD9**	3.10E-01	1.77E-04	4.65E-01	1.86E-03	3.34E-02	1.27E-01
16	**hsa-miR-19b-3p**	**LPHN2**	2.92E-01	1.91E-04	5.45E-01	6.61E-03	1.43E-01	1.05E-01
17	**hsa-miR-34a-5p**	**ATXN2L**	5.44E-01	2.08E-04	2.02E-02	1.87E-01	7.45E-02	9.04E-02
18	**hsa-miR-17-5p**	**CDKN1A**	7.13E-01	2.44E-04	5.07E-02	1.35E-01	2.31E-01	2.80E-02
19	**hsa-miR-222-3p**	**KIT**	6.70E-02	2.50E-04	1.39E-01	1.07E-01	5.30E-01	1.87E-04

20	**hsa-miR-19b-3p**	**ATM**	3.57E-01	2.54E-04	1.75E-01	2.31E-02	1.44E-01	1.87E-02
21	**hsa-miR-19b-3p**	**VAMP3**	3.98E-01	2.77E-04	1.33E-02	2.68E-01	7.61E-01	6.52E-04
22	**hsa-miR-19b-3p**	**NDEL1**	3.58E-01	2.83E-04	6.18E-01	1.40E-02	2.08E-01	7.53E-02
23	**hsa-miR-200a-3p**	**RIN2**	4.26E-01	3.18E-04	1.41E-02	1.02E-01	2.35E-01	1.92E-03
24	**hsa-miR-19b-3p**	**CCNL1**	1.55E-01	3.27E-04	7.41E-01	9.12E-04	8.17E-03	1.17E-01
25	**hsa-miR-19b-3p**	**CNOT6**	1.34E-01	4.64E-04	2.43E-01	8.03E-03	1.06E-01	1.44E-02
26	**hsa-miR-19b-3p**	**WDR68**	1.52E-01	4.65E-04	2.80E-01	4.53E-03	1.17E-01	2.31E-02
27	**hsa-miR-221-3p**	**ZNF192**	1.97E-01	4.69E-04	5.51E-01	1.33E-04	3.01E-02	4.25E-01
28	**hsa-miR-19b-3p**	**FAM62A**	4.45E-01	4.79E-04	2.21E-01	2.64E-02	3.96E-01	2.78E-02
29	**hsa-miR-221-3p**	**PANK3**	3.70E-01	5.77E-04	1.37E-01	1.86E-02	1.84E-01	1.59E-02
30	**hsa-miR-19b-3p**	**CPD**	4.21E-01	5.83E-04	1.67E-01	1.10E-01	6.43E-01	9.57E-03
31	**hsa-miR-34a-5p**	**HIST1H4I**	8.45E-01	5.88E-04	2.41E-01	3.94E-02	1.69E-02	3.32E-01
32	**hsa-miR-19b-3p**	**WEE1**	3.05E-01	6.13E-04	4.52E-02	1.19E-01	3.80E-01	2.24E-03
33	**hsa-miR-19b-3p**	**HOXA5**	3.35E-01	6.15E-04	8.19E-02	3.18E-02	4.54E-01	7.20E-03
34	**hsa-miR-19b-3p**	**SEMA4C**	1.64E-01	6.63E-04	1.57E-01	6.26E-02	7.63E-01	6.24E-03
35	**hsa-miR-19b-3p**	**FAM46A**	2.87E-01	6.65E-04	5.61E-02	6.18E-02	3.05E-01	2.57E-02

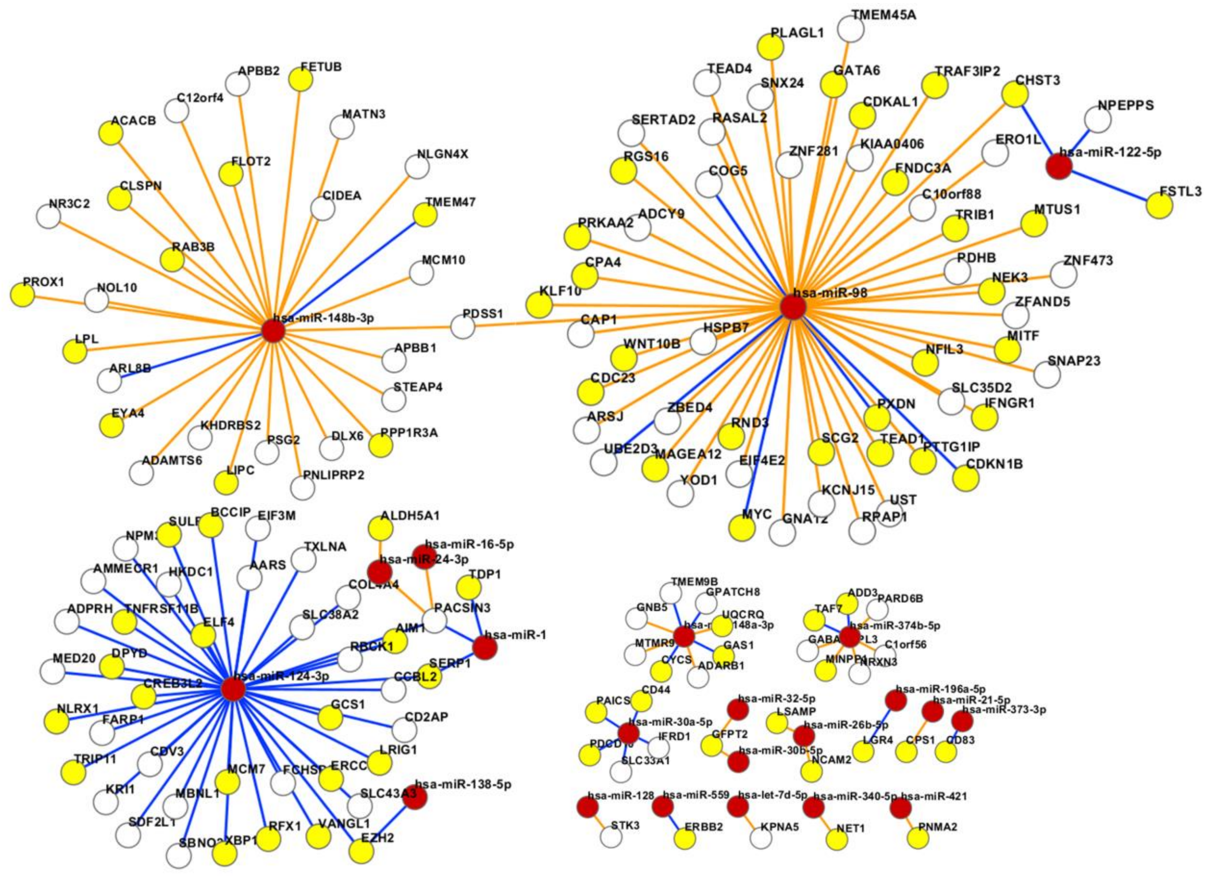

Supplemental Figure 1. Network visualization of all significant miRNA-mRNA interaction pairs in terms of clinical outcome in ovarian cancer patients.
